# Expression and function of histamine and its receptors in atopic dermatitis

**DOI:** 10.1186/s40348-015-0027-1

**Published:** 2015-12-21

**Authors:** M. Albrecht, A. M. Dittrich

**Affiliations:** Department for Pediatric Pneumology, Allergology and Neonatology, Hannover School of Medicine, Carl-Neuberg-Str. 1, 30625 Hannover, Germany

**Keywords:** Atopic dermatitis, Histamine, Histamine receptors, Immunology, Treatment

## Abstract

**Background:**

Atopic dermatitis constitutes a most burdensome chronic inflammatory skin disease. Standard treatment is cumbersome and often targets its main symptom, pruritus, only insufficiently.

**Findings:**

Recent advances in our understanding of the role of histamine and its four receptors suggest new approaches which target the histamine receptors alone or as combination therapies to more efficiently combat pruritus and inflammation in atopic dermatitis.

**Conclusions:**

With this review, we provide an overview on histamine and the expression of its four receptors on skin resident and nonresident cells. Furthermore, we summarize recent studies which suggest anti-histamine therapy to efficiently combat pruritus and inflammation in atopic dermatitis and discuss possible approaches to incorporate these findings into more effective treatment strategies for atopic dermatitis in childhood.

## Introduction

Atopic dermatitis (AD) is one of the most prevalent chronic diseases in early childhood. AD affects about 20 % of all children [1] and can prevail into adulthood. Its severe pruritus, stigmatizing local appearance, and relapsing course turn it into a chronic disease with a heavy psychological burden on the affected families. Treatment is cumbersome and time-consuming, and pruritus can only be addressed insufficiently, mainly by improvements in overall disease control which cannot always be achieved.

Immunologically, AD is driven by TSLP-secretion by keratinocytes and epithelial cells which modulate dendritic cell (DC) and basophil function to preferentially induce a Th2 immune response. The Th2 associated cyto- and chemokines IL-4, IL-5, IL-13, TARC/CCL17, and MDC/CCL22, in turn, lead to recruitment of mast cells and eosinophils whose mediator release includes histamine [2, 3].

Plasma histamine levels are higher in AD patients than in healthy controls [4], and histamine is detected readily in AD skin lesions [5]. Histamine’s pleiotropic actions were acknowledged since its discovery and since the 1960s attributed to the secretion by different cell types (Table 1) and the differential tissue expression of several receptors (Table 2, [6, 7]). Four histamine receptors (HRs) have been described; thus far, the “youngest” of these receptors, the H4-receptor was discovered in 2000 only [8, 9]. The first clinically used anti-histamine was synthesized in 1942, and ever since, anti-histamines have been a mainstay of anti-allergic, particularly anti-pruritic therapy mainly by targeting the H1R.

**Table 1:** Resident and nonresident cells of the skin capable of histamine secretion

Cell type	
Neurons [110]	
Basophils [111]	
Mast cells [112]	
Platelets [113]	
DCs [114]	
T cells [115]	
Macrophages [51]

**Table 2:** Expression of HRs in resident and nonresident cells of the skin

Cell type	Histamine receptor expression
Mast cells	H1R [10], H4R [116, 117]
Eosinophils	H1R [10], H2R, H3R [32, 91, 118, 119] H4R [120, 121]
Basophils	H4R [116, 117]
Neutrophils	H1R [10], H2R [122, 123], H4R [116, 117]
Dendritic cells	H1R [34], H2R [34], H3R [32, 19, 118, 119], H4R [33]
Langerhans cells	H4R [35]
Monocytes/macrophages	H1R [10], H2R [122, 123], H3R [32, 91, 118, 119], H4R [8]
T cells	H1R [10], H2R [122, 123], H4R [100, 117, 124]
B cells	H1R [10], H2R [122, 123]
Keratinocytes	H1R [10], H2R [71]
Endothelial cells	H1R [10], H2R [122, 123], H1R–H4R [74]
Smooth muscle cells	H1R [10], H2R [122, 123]
Fibroblasts	H1R [82]
Neurons	H1R [10], H2R [122, 123], H3R [32, 91, 118, 119]

### Targeting histamine as a therapeutic approach for AD

For a long time, histamine’s effects in allergic skin disease were thought to be mediated solely by its action on H1Rs. In the skin, H1Rs are expressed on vascular smooth muscle cells, endothelial cells, neurons, and different immune cells such as monocytes, neutrophils, DCs, and T and B cells where their activation drives the typical symptoms of immediate hypersensitivity responses, comprising edema and pruritus [10]. Anti-H1R antagonism has been a mainstay of anti-allergic treatment regimens since the advent of the first anti-H1R antagonists by chemical synthesis. Their efficiency as anti-pruritic agents in urticaria and inclusion in the therapy of anaphylaxis are undisputed. The effects of H1R blockade in anaphylaxis critically depend on its vasoconstrictive properties. The anti-pruritic effects are mediated by a reduction of histamine-dependent release of pruritogenic pro-inflammatory mediators such as bradykinins, serotonin, prostaglandins, and substance P by mast cells which all can confer an itching sensation. Moreover, histamine regulates the release of nerve growth factor [11] and semaphorin 3A from keratinocytes which also act as pruritic factors, and H1R targeting reduces IL-31, a pro-pruritic cytokine which additionally plays an important role in skin barrier integrity [12–14].

In addition to its pro-pruritic effect, several studies acknowledge a role for the H1R on immune cells in mediating inflammatory effects of allergic skin diseases with antagonism of the H1R by several drugs demonstrating anti-inflammatory properties in different experimental models of AD [15–19]. Thus, antagonism of histamine has the potential to affect pruritus as well as inflammation in AD. However, numerous clinical studies have shown that AD is only insufficiently addressed by anti-H1Rs [20, 21] since no significant reduction in pruritus or disease severity over control was observed with topical or systemic treatment [22, 23]. National guidelines thus do not recommend treatment with anti-H1R antagonists for the therapy of childhood AD anymore, neither systemically nor topically (AWMF S3 Leitlinie der deutschen dermatologischen Gesellschaft; [24]). However, as we will discuss below, new findings concerning the effect of histamine and HR expression in AD and AD models necessitate a re-thinking of the approaches used to target the histamine pathway in atopic skin disease and suggest new possibilities for anti-histamine therapy in AD.

### Histamine and histamine receptor expression in skin-resident and nonresident cells

In this review, we provide the readers with a comprehensive summary of histamine and histamine receptor expression. In this context, we would like to caution that, as usual, antibody-dependent methods to determine expression levels depend on the antibody-specificity of the antibodies used and thus need to be received with the necessary prudence. As outlined above, a variety of cell types within the skin are capable of histamine secretion (Table 1). In turn, histamine can act on a variety of cell types in the skin due to the widespread expression of its four receptors on a large variety of cell types in the skin (Table 2).

### Mast cells, eosinophils, and basophils

Quantitatively, mast cells and eosinophils are mainly responsible for histamine secretion in the skin. They can respond to histamine secretion due to the expression of different HRs (Tables 1 and 2). For eosinophils, differential migratory and anti-migratory effects of histamine have been described depending on its dose which differentially acts on H1Rs and H2Rs [25, 26]. Additionally, both eosinophil and mast cell migration into the skin are driven by H4Rs [26–28]. Basophils, similar to eosinophils and mast cells, show H4R-dependent chemotaxis [29]. Indeed, findings on the effects of histamine via H4Rs on mast cells and eosinophil migratory behavior were instrumental in suggesting that combination therapies with H1Rs and H4Rs could provide synergistic efficacy in atopic skin diseases.

### Neutrophils

In the context of allergic late-phase reactions, neutrophils also infiltrate the skin lesions in AD and are sought to contribute to disease flares [30]. While expression of H1R, H2R, and H4R has been described on neutrophils (Table 2), for many functional studies on the effect of histamine on neutrophils, it remains unclear whether these effects can be attributed to a direct effect of histamine on neutrophils or is mediated by effects of histamine on other cells types, for instance mast cells, which in turn affect neutrophil functions. Furthermore, histamine’s effects on neutrophils often show disparate results [31], suggesting that more research is needed to increase our understanding of histamine’s effects on neutrophils and potential beneficial aspects in the context of AD.

### Dendritic cells and langerhans cells and macrophages

In immune responses, histamine’s action on dendritic cells and, in the skin, Langerhans cells (LC)s), which express all four histamine receptors (DCs [32–34]) or the H4R (LCs [35]), warrants special attention. DCs can often be found in close proximity of degranulated mast cells and histamine secretion by mast cells controls DC migration to the lymph nodes [36]. Contact hypersensitivity (CHS) reactions depend on the interaction of mast cells and DCs [37], and the mast cell-DC interaction is also critical in mediating tolerance to autologous antigens in the skin [38]. Vanbervliet et al. showed earlier on that H1R signaling is an important mediator for the induction of allergen-dependent AD-like lesions in murine skin [39]. Several studies have shown that HR signaling critically shapes the T cell repertoire by modulation of DC effects on T helper (Th) cell polarization. For example, signaling via H1R on DCs promotes T helper type 1 (Th1) polarization whereas H2R signaling favors Th2 polarization and IL-10 secretion by DCs [40–44]. Furthermore, the same group showed that H1R signaling on DCs plays a critical role in the balance of IFN-γ and IL-17 secretion, driving an AD phenotype in mice [45]. Gschwandtner et al. showed that IFN-γ upregulates H4R expression on inflammatory DCs of AD patients and H4R stimulation of these cells leads to reductions in TNF-α and IL-12 secretion [46]. In a CHS model which displays some features of AD, blocking of the H4R led to decreased DC migration to the LNs, resulting in decreased Th2 and Th17 cytokine secretion [47, 48]. In summary, these findings point towards a decisive role for histamine in modulating DC/LC function to distinctively shape the T cell response in AD.

In contrast to the development of DCs, during macrophage differentiation from monocytes H1R is up- and H2R downregulated [49, 50]. Besides, macrophages are capable to produce and secrete histamine themselves [51]. Stimulation of lung macrophages by H1R leads to release of pro-inflammatory mediators like IL-6 [52], thereby amplifying an ongoing inflammatory response, a process which might also take place in the skin. Moreover, stimulation of the IgE specific receptor (FcεRI) on human monocytes promotes differentiation into H1R-expressing macrophages, which are pro-inflammatory and exhibit increased histamine secretion [53]. The same authors could prove that H1R-positive macrophages are present in AD lesions of the skin. Additionally, stimulation of the H4R increases chemotaxis and phagocytosis in murine bone marrow derived macrophages and a macrophage-like cell line [54] furthermore aggravating inflammation in AD. Antagonism of H1R and H4R might thus be a strategy to inhibit self-amplifying pro-inflammatory circles in skin macrophage function.

### T cells

Polarized T cells display differential HR expression, which contribute to the polarization and activation status by histamine; Th1 cells express higher levels of H1R, while T helper type II (Th2) cells express higher levels of H2R. H1R stimulation is necessary for optimal IFN-γ secretion [55, 56], increases Th1 proliferation, and has chemotactic effects on airway Th1 cells [57]. H1R knockout mice show enhanced secretion of Th2 cytokines and reduced severity of Th1-dependent autoimmune disease [58], pointing towards preferential Th1 activation by H1R. Moreover, triggering of H1R in allergic rhinitis patients increases Th2 cytokine secretion [59]. By resorting to differential H4R knockout mice, Hartwig et al. could elegantly show that via H4R signaling on DCs, the H4R is involved in T cell polarization towards a Th2 phenotype [60].

However, histamine’s effects on T cells are pleiotropic, depending on the HR addressed. For example, histamine exerts immunoregulatory effects via the H2R encompassing different Th cells: H2R knockout mice display upregulation of both Th1 and Th2 cytokines [55] and triggering of the H2R enhances TGF-β-dependent suppression of Th2 responses and proliferation [61], reduces IL-12 secretion [62], and can induce IL-10 secretion by Th2 cells [63]. These results assign a critical role to the H2Rs in promoting peripheral tolerance, which is supported by studies with cells from bee keepers tolerant to bee venom which show H2R-dependent suppression of IL-4 secretion by T cells in favor of increases in IL-10 secretion [64]. Resistance to H2R’s immunosuppressant properties and concomitant H1R-dependent Th2 activation as observed in patients with allergic rhinitis might underlie atopic disease manifestations and warrants further research [59].

Different studies also assign a role for histamine signaling on tolerance induction or reduction by direct HR activity on regulatory T cells (Tregs). In that line, triggering of the H1R on Tregs decreases their suppressive function which was associated with decreases in CD25 and FoxP3 expression [65] while H4R triggering stimulates Treg frequency [66] and migration and inhibits IL-12 and CCL2 secretion [67]. To complicate the picture even further, substantially, less is known on the expression of HRs on newer T helper cell subsets such as Th9, Th17, or Th22 cells, as well as possible functional consequences of such expression. H4R expression on Th17 has been described, and histamine stimulation of these cells increased IL-17 secretion and overall activation [68], suggesting that these cells might also be efficiently targeted by H4R antagonists.

### B cells

Histamine’s effects on B cells have not received much attention thus far and the few studies that address this question do not well discern direct or indirect effects of histamine on B cells as they did not resort to B cell-specific knockouts and could thus be mediated by histamine’s effects on T cells or DCs. In HR knockout models, histamine’s effects on B cells seem to largely depend on the requirement for T cell help. For T cell-independent B cell proliferation and immunoglobulin secretion, histamine, via H1R, appears to have a positive regulatory role while the production of T cell-dependent antigens is suppressed via the H1R. Abolishment of H2R signaling, however, decreases the production of T cell-dependent immunoglobulin secretion by B cells [69].

### Keratinocytes

Th polarization and differential activation of Th cells in the skin is also driven by HR expression on keratinocytes. Histamine increases the production of MCP-1, RANTES, and GM-CSF in keratinocytes [70], chemokines with known pro-inflammatory and Th2-promoting effects. Furthermore, H1R expression on keratinocytes has been shown to differentially regulate the production of Th1 vs. Th2 chemokines, providing a negative-feedback signal for an existing Th2-dominant inflammation by enhancing Th1 supporting chemokines and suppressing Th2 favoring chemokine secretion [71]. Histamine also has effects on other important keratinocyte functions which go awry in AD: it decreases the formation of tight junctions and the expression of filaggrin, processes essential for the maintenance of skin barrier function, and increases keratinocyte proliferation, leading to hyperkeratosis [72, 73].

### Endothelial cells

One main action of histamine on endothelial cells is its role in increasing vascular permeability by inducing endothelial barrier dysfunction contributing to pathological processes like anaphylactic reactions. All four identified receptors (H1R–H4R) can be found on endothelial cells of dermal origin showing different subcellular distribution amongst the receptors [74]. The disruption of endothelial barrier function by histamine, however, seems to be mediated primarily through H1R via the small GTPase RhoA and its associated kinase ROCK, as treatment with a ROCK inhibitor protects from anaphylactic shock in experimental models [75]. Another function of histamine is upregulation of P-selectin on endothelial cell of dermal origin, enabling recruitment of leukocytes and thus enhancing inflammation [76]. Endothelial cells can amplify the immune response by secretion of IL-6 and IL-8 [77, 78] and upregulation of TLR2 and TLR4 after activation with histamine [79]. Although the impact of histamine actions on endothelial cells in AD pathology has been described as minor, data describing a longer lasting change in epithelial barrier function due to histamine treatment in endothelial cells of dermal origin compared to endothelial cells from the umbilical vein or cardiac origin [74] might indicate a more important role. Moreover, histamine seems to have a pro-angiogenetic effect, since H1R signaling on umbilical cord endothelial cells promotes bFGF (basic fibroblast growth factor)-induced VEGF (vascular endothelial growth factor) secretion and in turn proliferation and tube formation [80].

### Smooth muscle cells/fibroblasts

Besides the well-known involvment of histamine in the weal and flare reaction by acting on vascular smooth muscle cells via H1R [81], recent evidence exists that histamine can influence skin fibroblast differentiation into myofibroblasts and thus play a role in fibrotic events: Histamine inhibits TGF-beta-mediated expression of αSMA (α-smooth muscle actin) by fibroblasts. TGF-beta, however, induces downregulation of H1R on fibroblasts leading to a balance between TGF-beta-mediated and histamine-mediated actions [82]. More evidence for an important role of histamine in the fibrotic processes in the skin gives the work by Yang et al. who could show that histamine induces periostin (a profibrotic protein) production by primary dermal fibroblasts in an ERK1/2-mediated manner by activation of H1R [83]. An increased expression of periostin in lesional skin of AD patients is indeed described [84], rendering it possible that antagonism of H1R might influence histamine-induced tissue remodeling in AD.

### Neurons

Specific neurons in the skin mediate histamine-induced itching sensations, mediated by specific conducting pathways [85, 86]. Those skin innervating sensory neurons express the H1R [87], H3R, and H4R, and activation of H1R and H4R promotes pruritus further enhanced by H3R antagonism [88]. Histamine-induced pruritic signals are mediated via protein kinase Cδ [89] and activation of TRPV1 (a nonselective catione channel) via activation of phospholipase A [90]. Also, in vivo and in vitro studies suggest that the H3R is involved in mediating neuro-inflammatory effects by neuro-immune interactions of nerves and T cells or mast cells [91, 92].

### New histamine-directed approaches

The expression and effects of histamine-mediated signaling on skin-resident and nonresident cells, as discussed above, beg to re-evaluate the efficacy of histamine targeting in AD. Particularly, new studies which closer delineate the main cellular players in AD, their histamine-responsiveness, and findings on the most recently discovered HR, the H4R, suggest that novel targeting strategies incorporating the H4R could be effective in targeting not only pruritus but also inflammation in this abundant pediatric skin disease.

In that line, targeting the H4R has particular appeal due to its pro-Th2 effects. H4R antagonism, via modulation of DC activation, reduces Th2-driven airway inflammation [47, 48, 93]. Similarly, the H4R mediates Th2-dependent skin inflammation [47, 48] with H4R antagonists reducing TARC secretion by mast cells, thereby reducing Th2 activation and polarization [19]. Signaling via H4R is also critically implied in a hapten-induced AD model [94]. These studies translate into other findings, showing that H4R antagonists are more efficient than other HR antagonists in suppressing allergen-induced pruritus [95] and H4R targeting has been shown to efficiently target inflammation and pruritus in different model systems [96–99], with the H4Rs anti-pruritic effect possibly relying on reduction of IL-31 secretion [100]. Furthermore, the H4R has been shown to mediate Th17-dependent inflammation in arthritis [101], suggesting that Th17-driven inflammation which has been associated with AD [102–105], particularly in acute skin lesions [106], can also be efficiently countered by H4R antagonists. The first clinical studies with JNJ39758979, a selective H4R antagonist, showed good clinical efficacy in reducing histamine-induced pruritus in healthy subjects [107] as well as in reducing itch severity and duration in patients with AD [108]. Unfortunately, severe side effects occurred in two patients with this compound in the phase II trial, leading to termination of the study.

Combination therapy with H1R and H4R antagonists might be an even more powerful approach. In a T cell transfer model, the use of specific H1- and H4-receptor antagonists revealed a crucial role for H1- and H4-receptors for Th2 migration and cytokine secretion in a Th2-driven model of skin inflammation. While H1- and H4-receptor antagonists both reduced Th2 recruitment to the site of challenge, local cytokine responses in skin-draining lymph nodes were only reduced by the combined application of H1- and H4-receptor antagonists [109]. These results might explain why antagonism of H1R alone had no significant effects on the dermatitis in an AD model [97], but co-administration did affect inflammation as well as pruritus [19]. Dunford et al. demonstrated superiority of targeting the H4R for pruritus compared to blockade of H1R. However, in H4R knockout mice, they showed an additive effect of H1R antagonism on pruritus, results which also argue for a combination approach to achieve the most potent suppression of histamine’s effects [95].

## Conclusions

Taken together, a large number of in vitro and animal studies suggest combined H1R/H4R targeting to successfully address pruritus and inflammation, two closely inter-related symptoms of AD. Initial clinical trials with an H4R antagonist did show good efficacy with regard to pruritus, yet the side-effects encountered prevent its development as a marketable drug. Further improvements in chemical compounds targeting the H4R are thus needed to assess the effect of H4R treatment alone or in conjunction with H1R antagonists on allergic skin inflammation and, provided efficacy, ultimately take them into clinical trials to assess their clinical potential in a disease which would clearly benefit from new therapeutic approaches.

## Abbreviations

AD: atopic dermatitis
CHS: contact hypersensitivity
HR: histamine receptor
Th: T helper cell
Th1: T helper type I cell
Treg: regulatory T cell
